# Peptidylarginine deiminase 4-mediated citrullination in human disease: molecular mechanisms and therapeutic targeting

**DOI:** 10.3389/fphar.2026.1854078

**Published:** 2026-05-15

**Authors:** Ping Zou, Xiao-dan Su, Wei Tian, Xiang-yang Xie, Ke Yang

**Affiliations:** 1 Department of Pharmacy, The Central Hospital of Wuhan, Tongji Medical College, Huazhong University of Science and Technology, Wuhan, China; 2 Department of Pharmacy, General Hospital of Central Theater Command, Wuhan, China; 3 College of Nursing and Health Management & College of Life Science and Chemistry, Wuhan Donghu University, Wuhan, China

**Keywords:** autoimmune disease, cancer, citrullination, inflammation, neutrophil extracellular traps, PAD4 inhibition, peptidylarginine deiminase 4, thrombosis

## Abstract

Peptidylarginine deiminase 4 (PAD4) is a calcium-dependent enzyme that catalyzes protein citrullination, a post-translational modification that can alter protein charge, conformation, and function. Among the mammalian peptidylarginine deiminase isoforms, PAD4 is distinguished by its nuclear localization, its ability to citrullinate histones, and its established roles in chromatin remodeling, transcriptional control, and neutrophil extracellular trap formation. Through these activities, PAD4 links epigenetic regulation with innate immunity, inflammation, and thrombosis. Dysregulated PAD4 activity has been implicated in rheumatoid arthritis systemic lupus erythematosus central nervous system demyelinating disease, cancer, atherosclerosis, acute thrombotic syndromes, and sepsis. Mechanistically, PAD4-mediated citrullination influences chromatin accessibility, inflammatory signaling, autoantigen generation, extracellular matrix remodeling, and immunothrombosis. These observations have made PAD4 an attractive pharmacological target. Drug development has progressed from early irreversible inhibitors, including Cl-amidine and F-amidine, to more selective reversible compounds such as GSK484 and GSK199, as well as newer orally available candidates such as JBI-589 and JBI-1044. Emerging approaches, including allosteric ligands, cyclic peptides, and functional antibodies, continue to broaden the PAD4-targeting landscape. In this review, we summarize the molecular basis of PAD4-mediated citrullination, examine its contributions to major disease settings, evaluate current and emerging PAD4-directed therapies, and discuss the key challenges that must be addressed for successful clinical translation.

## Graphical Abstract

**Figure:**
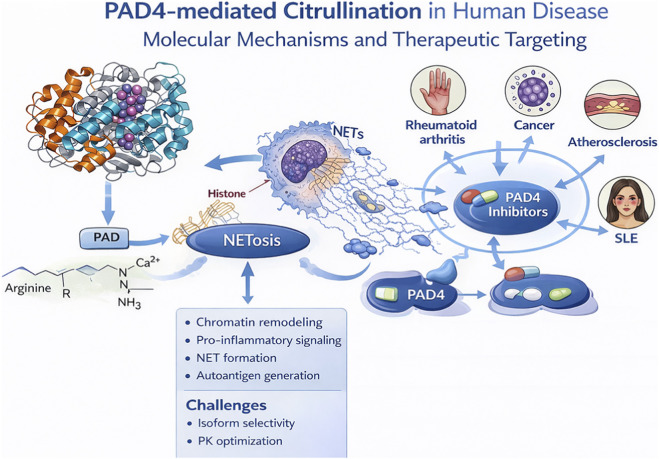

## 1 Introduction

Post-translational modifications (PTMs) are essential determinants of protein function and are fundamental to the dynamic regulation of cellular physiology. By introducing chemical changes to specific amino acid residues, PTMs greatly expand the structural and functional diversity of the proteome beyond the constraints imposed by the genome. Major PTMs, including phosphorylation, acetylation, methylation, ubiquitination, glycosylation, and citrullination, modulate protein stability, localization, intermolecular interactions, catalytic activity, and signaling output. Through these mechanisms, PTMs participate in the control of gene expression, cell-cycle progression, metabolism, stress adaptation, immune activation, and cell fate decisions. Unsurprisingly, the dysregulation of PTM networks has been increasingly recognized as a central contributor to many human diseases, including autoimmune disorders, cardiovascular pathology, neurodegeneration, and cancer (Venne et al., 2014; Millán-Zambrano et al., 2022; Aletta and Hu, 2008; Karve and Cheema, 2011).

Among these modifications, protein citrullination, also termed deimination, has emerged as a distinctive PTM with broad implications for epigenetic regulation and immune biology. Citrullination is the calcium-dependent conversion of positively charged arginine residues into neutrally charged citrulline residues, resulting in altered hydrogen-bonding properties, reduced electrostatic interactions, and modified protein conformation and function (Zhang X. et al., 2024; Yu and Proost, 2022; Bicker and Thompson, 2013). Although the chemical change is subtle, its biological consequences can be profound. By neutralizing arginine side chains, citrullination can weaken protein-protein and protein-nucleic acid interactions, reshape protein folding, alter complex assembly, and influence protein degradation. In the nucleus, histone citrullination can promote chromatin decondensation and reprogram transcriptional accessibility (Wang et al., 2004). Outside the nucleus, citrullination affects structural proteins, extracellular matrix components, and immune mediators, thereby influencing tissue architecture, autoantigenicity, and inflammatory responses (Bashir et al., 2025; Jonsson et al., 2020; Wu et al., 2023; Thiam et al., 2020a).

Citrullination is catalyzed by the peptidylarginine deiminase (PAD) family, which comprises five mammalian isoforms: PAD1, PAD2, PAD3, PAD4, and PAD6 (Aletta and Hu, 2008; Yu and Proost, 2022; Bicker and Thompson, 2013; Jones et al., 2009). These isoforms exhibit distinct tissue distribution, substrate preference, subcellular localization, and physiological function. Among them, PAD4 occupies a unique position because it contains nuclear localization signals and can directly citrullinate histones and chromatin-associated substrates (Wang et al., 2004; Hagiwara et al., 2005). PAD4 is expressed predominantly in cells of the innate immune system, especially neutrophils and macrophages, where it integrates calcium signaling, inflammatory activation, and transcriptional responses. One of its best characterized functions is its role in neutrophil extracellular trap (NET) formation, in which PAD4-mediated histone citrullination contributes to chromatin decondensation and extracellular release of DNA-protein complexes that entrap pathogens (Thiam et al., 2020b; Lewis et al., 2015; Papayannopoulos, 2018; Cuthbert et al., 2004). Although all PAD isoforms catalyze citrullination, PAD4 is uniquely defined by its nuclear localization, histone-directed activity, and dominant role in NET formation; the following mechanistic discussions therefore focus on PAD4-specific functions unless general PAD family properties are explicitly noted.

The biological significance of PAD4 is best understood through a limited set of recurring mechanisms rather than through repeated disease-by-disease descriptions. Across autoimmune, malignant, vascular, and infectious settings, dysregulated PAD4 promotes citrullinated neoantigen formation, PAD4-dependent NETosis and immunothrombosis, chromatin/transcriptional remodeling, and context-dependent inflammatory amplification (Suzuki et al., 2003; Lood et al., 2016; Knight et al., 2014; Rosell et al., 2022; Li et al., 2010). These shared mechanisms explain why PAD4 is implicated in rheumatoid arthritis (RA), systemic lupus erythematosus, cancer, cardiovascular disease, and sepsis, while also emphasizing that its contribution varies with cell type, tissue environment, and disease stage.

These disease associations have transformed PAD4 from a mechanistic curiosity into a pharmacologically relevant target. Early proof-of-concept studies demonstrated that inhibition of PAD4 suppresses histone citrullination, reduces NET formation, attenuates inflammatory and thrombotic responses, and ameliorates disease phenotypes in experimental models (Lewis et al., 2015; Knight et al., 2014). More recent medicinal chemistry efforts have produced increasingly selective and potent reversible inhibitors, improved orally available compounds, and alternative targeting approaches such as antibodies, peptides, and allosteric ligands (Aiken et al., 2025). Collectively, these developments indicate that PAD4 is not merely biologically important but also chemically tractable.

At the same time, translational development remains complex. PAD4 shares substantial structural homology with other PAD isoforms, making isoform selectivity difficult to achieve (Funabashi et al., 2021). Because PAD4 contributes to host defense, excessive or prolonged inhibition could compromise antimicrobial immunity. Moreover, many currently available inhibitors remain limited by suboptimal pharmacokinetics, incomplete *in vivo* selectivity, or insufficient biomarker support for patient stratification. Thus, although PAD4 represents an attractive therapeutic target, successful clinical translation will require a deeper understanding of its context-specific biology and more refined drug-development strategies.

In this review, we summarize the molecular and cellular basis of PAD4-mediated citrullination, discuss how PAD4 contributes to major disease settings, and assess current and emerging strategies for therapeutic targeting. Particular emphasis is placed on issues that are likely to determine translational success, including isoform selectivity, biomarker development, pharmacokinetic optimization, and preservation of host defense.

## 2 Molecular and cellular basis of PAD4-Mediated citrullination

### 2.1 Citrullination as a post-translational modification

**FIGURE 1:**
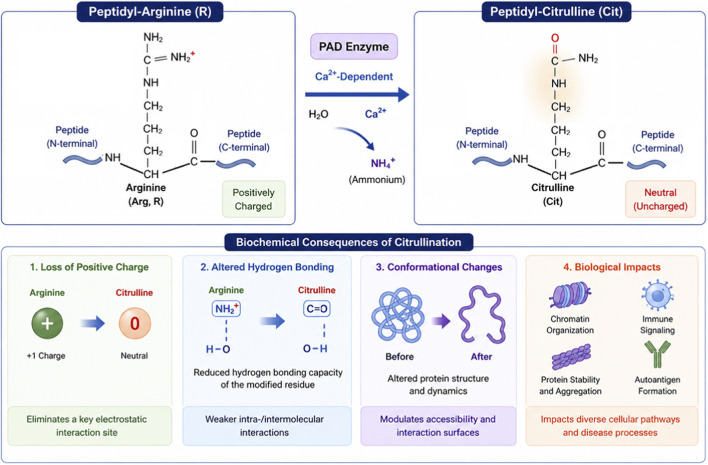
PAD-catalyzed conversion of peptidyl-arginine to peptidyl-citrulline.

Protein citrullination is a hydrolytic PTM in which the imino group of arginine is converted to a ketone-like ureido group, yielding citrulline and eliminating one positive charge from the modified residue (Zhang X. et al., 2024; Yu and Proost, 2022; Bicker and Thompson, 2013). This conversion has important consequences for the physicochemical properties of proteins (Figure 1). Arginine is strongly basic and often participates in electrostatic interactions with negatively charged molecules such as nucleic acids, phospholipid head groups, sulfated glycans, and acidic protein surfaces. Conversion to citrulline weakens these interactions, alters local hydrogen-bonding networks, and may reshape tertiary or quaternary protein structure. As a result, citrullination can influence substrate binding, complex assembly, phase behavior, enzymatic activity, and susceptibility to proteolysis (Zhang X. et al., 2024; Yu and Proost, 2022; Bicker and Thompson, 2013; Bashir et al., 2025).

Unlike phosphorylation or acetylation, citrullination is generally regarded as functionally irreversible under physiological conditions, because no dedicated “decitrullinating” enzyme has been established. Consequently, the biological consequences of citrullination are typically resolved through protein turnover, replacement, or cellular remodeling rather than direct reversal (Zhang X. et al., 2024). This feature may contribute to the long-lived immunological and structural effects of aberrant citrullination in chronic inflammatory disease.

The impact of citrullination depends not only on the identity of the modified substrate but also on the location, extent, and cellular context of the modification. In some proteins, citrullination alters activity directly; in others, it changes interactions with partner proteins or nucleic acids. In structural proteins such as vimentin, fibrin, or myelin basic protein (MBP), citrullination can alter filament assembly, mechanical stability, or membrane interaction (Bashir et al., 2025; Jonsson et al., 2020; Moscarello et al., 2002; Martinsen and Kursula, 2022; Yan et al., 2016). In histones, citrullination loosens chromatin architecture and modulates access of transcription factors and chromatin-remodeling complexes (Wang et al., 2004; Cuthbert et al., 2004; Christophorou et al., 2014; Leshner et al., 2012; Arita et al., 2004; Knuckley et al., 2010; Zhou et al., 2018). In immune contexts, citrullination can generate neoepitopes and promote the formation of autoantigens recognized by pathogenic autoantibodies (Suzuki et al., 2003; Curran et al., 2020; Chen et al., 2024).

PAD enzymes catalyze the calcium-dependent hydrolytic deimination of peptidyl-arginine residues to generate peptidyl-citrulline. This reaction results in loss of a positive charge, altered hydrogen-bonding capacity, and changes in protein conformation and intermolecular interactions. These biochemical consequences underlie the effects of citrullination on chromatin organization, immune signaling, structural protein stability, and disease-associated autoantigen formation.

### 2.2 The PAD family and the unique features of PAD4

The PAD enzyme family consists of five calcium-dependent isozymes with overlapping but distinct patterns of expression and biological function (Aletta and Hu, 2008; Yu and Proost, 2022; Bicker and Thompson, 2013; Jones et al., 2009). PAD1 is most prominently expressed in epidermal and uterine tissues (Zhang et al., 2016), PAD2 is widely distributed and particularly abundant in muscle, brain, and immune tissues (Yu and Proost, 2022), PAD3 is associated mainly with hair follicles and epidermis, PAD4 is enriched in granulocytes and other immune cells (Bicker and Thompson, 2013), and PAD6 is expressed largely in oocytes and early embryos, although its activity remains unconfirmed (Ranaivoson et al., 2024).

PAD4 differs from the other PADs in several biologically important ways. First, it contains canonical nuclear localization signals that enable active nuclear accumulation (Arita et al., 2004; Knuckley et al., 2010). Second, it can directly citrullinate histones and chromatin-associated substrates, placing it in direct contact with gene-regulatory machinery (Wang et al., 2004; Hagiwara et al., 2005; Cuthbert et al., 2004; Christophorou et al., 2014; Leshner et al., 2012). Third, PAD4 has a particularly well-established role in NET formation, where histone citrullination contributes to chromatin decondensation and extracellular DNA release (Thiam et al., 2020a; Lewis et al., 2015; Leshner et al., 2012; Clark et al., 2018). Fourth, PAD4 is strongly linked to autoimmune disease susceptibility and autoantigen generation, especially in rheumatoid arthritis (Suzuki et al., 2003; Curran et al., 2020; Kimura et al., 2023; Thomas et al., 2024; Pollmann et al., 2012; Zhang G. et al., 2024). Together, these features make PAD4 the PAD isoform most clearly connected to inflammation, immunothrombosis, and pharmacological intervention.

The human PADI4 gene is located on chromosome 1p36.13 (Suzuki et al., 2003). Genetic studies have identified polymorphisms associated with altered disease susceptibility, particularly in rheumatoid arthritis and, in some populations, systemic lupus erythematosus (Suzuki et al., 2003; Zhang G. et al., 2024; Geng et al., 2023). While the strength of these associations differs across ethnic groups, they support the notion that genetically influenced variation in PAD4 expression or function can shape disease risk and phenotype.

### 2.3 PAD4 structure and catalytic mechanism

**FIGURE 2:**
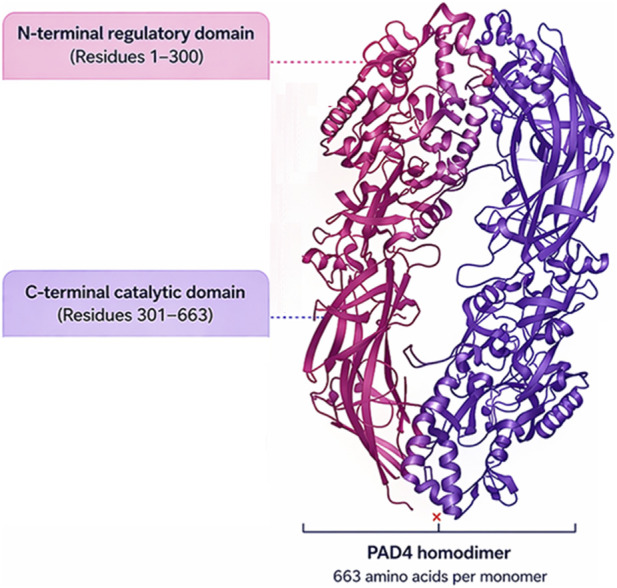
Structural organization of PAD4.

PAD4 is a 663-amino-acid enzyme that functions as a homodimer and contains an N-terminal regulatory domain and a C-terminal catalytic domain (Arita et al., 2004). The N-terminal region includes motifs important for protein-protein interactions and nuclear localization, whereas the C-terminal domain contains the active site responsible for catalysis (Figure 2). Structural studies have shown that PAD4 undergoes pronounced conformational changes upon calcium binding, and these rearrangements are essential for enzymatic activation (Arita et al., 2004; Zhou et al., 2018).

Calcium is a critical regulator of PAD4 function. Multiple calcium ions bind to PAD4 and stabilize an active conformation in which the catalytic cleft is properly assembled and substrate access is enabled (Arita et al., 2004; Zhou et al., 2018). In the absence of sufficient calcium, PAD4 remains in a less active or inactive conformation. This requirement helps explain why PAD4 is activated under conditions of cellular stress, inflammatory signaling, membrane disruption, or strong stimulation that perturbs intracellular calcium homeostasis. Bicarbonate has also been reported to enhance PAD activity under some experimental conditions, suggesting that PAD4 regulation depends not only on calcium concentration but also on the broader physicochemical milieu (Zhou et al., 2018).

The catalytic mechanism of PAD4 is thiol dependent and centered on the active-site cysteine residue Cys645 (Arita et al., 2004; Luo et al., 2006a). The catalytic cysteine performs nucleophilic attack on the guanidinium carbon of arginine, initiating hydrolysis and leading to the release of ammonia and formation of citrulline. Histidine and aspartate residues in the active site contribute to proton transfer and stabilization of the reaction intermediate. This catalytic arrangement has practical significance for drug development because the reactive cysteine can be targeted by covalent inhibitors, while the conformational dependence of the catalytic pocket creates opportunities for reversible and allosteric inhibition.

Beyond classical calcium-dependent regulation, recent evidence indicates that glycosaminoglycans (GAGs) such as heparin, heparan sulfate, and chondroitin sulfates can function as allosteric co-activators of PAD4 (Bereta et al., 2025). These negatively charged polysaccharides bind PAD4 with nanomolar affinity and markedly enhance its calcium sensitivity, lowering the half-maximal activation calcium concentration from ∼300 to 400 μM–∼100 μM or below. This effect depends on GAG chain length and negative charge density and requires PAD4 dimerization. Cryo-electron microscopy structures of PAD4-GAG complexes reveal that GAG chains bridge PAD4 dimers via dispersed positively charged surfaces, forming higher-order assemblies that involve the nuclear localization signal region and the vicinity of the calcium-binding sites Ca3–Ca5. These findings add a physiologically relevant layer to PAD4 regulation, as GAGs are abundant in the extracellular matrix and on cell surfaces, and their levels correlate with rheumatoid arthritis severity. Accordingly, GAG-mediated PAD4 activation may be particularly important in inflammatory microenvironments such as the rheumatoid joint, where both GAG release and PAD4 liberation from infiltrating neutrophils converge.

Although the catalytic core is relatively conserved across PAD isozymes, subtle structural differences can influence substrate preference, conformational dynamics, and inhibitor recognition (Bicker and Thompson, 2013; Jones et al., 2009; Aiken et al., 2025; Knuckley et al., 2010). Crystal structures have now been determined for PAD1 (Saijo et al., 2016), PAD2 (Slade et al., 2015), PAD3 (Funabashi et al., 2021), PAD4 (Arita et al., 2004), and PAD6 (Ranaivoson et al., 2024), revealing isoform-specific variations in active-site architecture, calcium-binding stoichiometry and coordination, dimerization mode (monomeric PAD1 vs. dimeric PAD2–PAD6), and regulatory loop dynamics that together provide a structural basis for the functional differences among PAD isozymes. For example, interactions involving residues that are not fully conserved among PAD family members have been exploited to improve PAD4 selectivity in medicinal chemistry campaigns (Aiken et al., 2025). Thus, the structural biology of PAD4 is directly relevant to the rational design of isoform-selective inhibitors.

PAD4 contains an N-terminal regulatory region and a C-terminal catalytic domain that harbors the active-site cysteine residue (Arita et al., 2004).

### 2.4 Substrate specificity and determinants of citrullination

PAD4 does not citrullinate all arginine-containing proteins equally. Substrate selection is influenced by several factors, including protein conformation, solvent accessibility of arginine residues, local amino acid context, subcellular localization, and the intensity and duration of PAD4 activation (Zhang X. et al., 2024; Bicker and Thompson, 2013; Knuckley et al., 2010). The accessibility of substrate arginine residues is especially important. Arginines buried within tightly folded domains are less likely to be modified than those positioned in flexible loops, disordered regions, histone tails, or partially unfolded states.

Histones are among the best characterized PAD4 substrates. PAD4 can citrullinate several arginine residues on histone H3, including H3R2, H3R8, H3R17, and H3R26, as well as sites on other histones under certain conditions (Wang et al., 2004; Hagiwara et al., 2005; Cuthbert et al., 2004; Christophorou et al., 2014; Leshner et al., 2012). Because histone tails are structurally accessible and rich in regulatory PTMs, they are particularly susceptible to signal-dependent modification. Histone citrullination has major consequences for chromatin organization and transcriptional control, discussed in detail below.

PAD4 also targets non-histone substrates relevant to immune regulation and disease. Citrullination of vimentin, fibrinogen, α-enolase, collagen, fibronectin, and other structural or extracellular proteins may alter antigenicity, assembly, protease sensitivity, and matrix-cell interactions (Bashir et al., 2025; Jonsson et al., 2020; Curran et al., 2020; Chen et al., 2024; Thomas et al., 2024; Cappelli et al., 2024; Shi et al., 2020; Gorzeń et al., 2025; Wang et al., 2022). In immune cells, PAD4 has been reported to modify signaling and transcriptional regulators, including components of the nuclear factor kappa B (NF-κB) pathway (Sun et al., 2017). Such modifications may influence nuclear translocation, DNA binding, or transcriptional competence and thereby amplify inflammatory gene expression.

Because substrate selection is context dependent, the biological outcome of PAD4 activation differs markedly between tissues and disease settings. In neutrophils, chromatin is a dominant substrate during NETosis. In synovial tissue, extracellular and intracellular proteins relevant to autoantigen generation become important. In tumors, both chromatin and extracellular matrix components may be modified. This context dependency is a key reason why PAD4 inhibition can have pleiotropic but disease-relevant effects.

A recent study using the Hybrid Combinatorial Substrate Library (HyCoSuL) approach, which incorporates both natural and over 100 unnatural amino acids, systematically profiled the substrate preferences of PAD2 and PAD4 and developed isoform-selective fluorogenic peptide substrates (Gorzeń et al., 2025). PAD4 displayed a strict preference for aspartic acid at the P2 position, whereas PAD2 accommodated a much broader range of residues at this site. By exploiting these differences and incorporating unnatural amino acids, substrates with high selectivity for either PAD2 or PAD4 were generated and validated in biochemical and cellular assays. These tools enabled the dissection of PAD2-versus PAD4-dominant activity in macrophage lysates and revealed that PAD2 is the major contributor to citrullination events in activated THP-1 macrophages. In addition, PAD4-mediated citrullination of vimentin was shown to modulate its susceptibility to calpain-1 cleavage and to diminish its function as a damage-associated molecular pattern. This work provides a powerful framework for isoform-specific activity profiling and may guide the design of selective inhibitors and activity-based probes targeting individual PAD isoforms.

These substrate-profiling data also provide a practical bridge between enzymology and drug discovery, because sequence- and residue-level preferences can be used to design more selective activity probes, competitive assays, and inhibitor scaffolds that discriminate PAD4 from the more permissive PAD2 isoform.

### 2.5 Functional consequences of PAD4-mediated citrullination

The functional consequences of PAD4-mediated citrullination can be broadly grouped into four categories: regulation of chromatin structure, modulation of signal-dependent transcription, remodeling of structural or extracellular proteins, and generation of immunogenic neoepitopes.

First, citrullination alters charge-based interactions within chromatin. Histones are enriched in positively charged lysine and arginine residues that bind negatively charged DNA. Citrullination reduces positive charge and weakens histone-DNA interactions, thereby promoting chromatin relaxation and changes in accessibility (Wang et al., 2004; Cuthbert et al., 2004; Christophorou et al., 2014; Leshner et al., 2012). In some contexts, this favors transcriptional activation; in others, it cooperates with other chromatin changes to repress specific loci.

Second, citrullination can modify signaling proteins and transcription factors, thereby influencing inflammatory and stress-response pathways. PAD4-mediated citrullination of NF-κB p65, for example, has been linked to altered nuclear localization and increased expression of inflammatory cytokines (Sun et al., 2017). This provides a direct biochemical link between a PTM enzyme and transcriptional amplification of immune responses.

Third, PAD4 can alter the properties of structural and extracellular proteins. Citrullination of matrix proteins may weaken tissue integrity, facilitate cell migration, or create a more permissive environment for inflammation and metastasis (Bashir et al., 2025; Jonsson et al., 2020; Shi et al., 2020; Wang et al., 2022; Tomita et al., 2025). Citrullination of cytoskeletal proteins may influence filament organization and cellular plasticity. These effects are especially relevant in chronic inflammatory tissue remodeling and in tumor dissemination.

Fourth, citrullination can create neoepitopes that are recognized by the adaptive immune system. This is particularly relevant in rheumatoid arthritis, where antibodies against citrullinated proteins are highly specific and clinically important (Suzuki et al., 2003; Pollmann et al., 2012; Salemme et al., 2019; Baños-Hernández et al., 2017). In this context, PAD4 functions not only as an enzyme but also as an upstream driver of autoantigen generation and immune propagation.

Collectively, these functional outputs explain why PAD4 exerts such broad influence over inflammatory, autoimmune, thrombotic, and neoplastic processes (Table 1). The enzyme does not act through a single pathway; rather, it reshapes multiple layers of cellular regulation, from chromatin architecture to extracellular immunogenicity.

**TABLE 1:** Major biological functions of PAD4 and representative substrates.

Biological process	Representative PAD4 substrates	Major functional consequence	Disease relevance
Histone citrullination and chromatin remodeling	Histone H3 arginine residues, including H3R2, H3R8, H3R17, and H3R26	Reduced positive charge on histones, chromatin decondensation, altered transcriptional accessibility, crosstalk with arginine methylation	Inflammation, immune activation, cancer, NETosis (Wang et al., 2004; Cuthbert et al., 2004; Christophorou et al., 2014; Leshner et al., 2012; Arita et al., 2004; Knuckley et al., 2010; Zhou et al., 2018)
Regulation of transcriptional signaling	Histones; NF-κB p65 and potentially other transcriptional regulators	Modulation of inflammatory gene expression, enhancement of cytokine production, context-dependent transcriptional activation or repression	Autoimmune disease, infection, chronic inflammation, cancer (Sun et al., 2017; Santos B et al., 2026; Nakashima et al., 1999; Knight et al., 2013)
NET formation	Histones during neutrophil activation	Chromatin decondensation, nuclear swelling, extracellular release of DNA-protein networks, antimicrobial trapping	Host defense, RA, SLE, thrombosis, atherosclerosis, sepsis, cancer-associated thrombosis (Thiam et al., 2020b; Papayannopoulos, 2018; Rosell et al., 2022; Clark et al., 2018; Liu et al., 2025)
Autoantigen generation	Fibrinogen, vimentin, α-enolase, collagen, citrullinated histones	Formation of neoepitopes, enhanced antigen presentation, autoantibody production	RA, SLE and other autoimmune disorders (Curran et al., 2020; Kaplan, 2025)
Extracellular matrix remodeling	Collagen, fibronectin and other matrix-associated proteins	Altered matrix integrity, enhanced cell migration and invasion, metastatic niche formation	Cancer invasion and metastasis; tissue remodeling in chronic inflammation (Shi et al., 2020; Wang et al., 2022; Tomita et al., 2025)
Immunothrombosis	Histones, NET-associated proteins, possibly anticoagulant proteins such as antithrombin	Platelet activation, fibrin deposition, endothelial injury, amplification of thrombin generation	Acute coronary syndromes (ACS), atherosclerosis, venous thrombosis, cancer-associated thrombosis, sepsis (Rosell et al., 2022; Fuchs et al., 2010; McDonald et al., 2017; Döring et al., 2017)
Myelin-related citrullination	Myelin basic protein	Reduced membrane interaction, destabilization of myelin architecture, increased susceptibility to degradation	multiple sclerosis and central nervous system demyelinating disease (Moscarello et al., 2002; Martinsen and Kursula, 2022; Yan et al., 2016; Geng et al., 2023)

PAD4 mediates citrullination at multiple cellular levels—chromatin, signaling factors, matrix proteins, and immune epitopes—which explains its broad involvement across inflammatory, autoimmune, thrombotic, and neoplastic diseases.

## 3 PAD4 in chromatin regulation, transcriptional control, and innate immunity

### 3.1 PAD4 as an epigenetic regulator

One of the defining features of PAD4 biology is its capacity to regulate chromatin directly. Because PAD4 localizes to the nucleus and targets histones, it occupies a unique position among the PAD family as a *bona fide* epigenetic modulator (Wang et al., 2004; Hagiwara et al., 2005; Cuthbert et al., 2004; Christophorou et al., 2014; Leshner et al., 2012; Arita et al., 2004). Histone tails are densely decorated with PTMs that collectively regulate nucleosome dynamics, chromatin compaction, and the recruitment of effector proteins. Within this highly integrated PTM landscape, citrullination can exert powerful structural and signaling effects.

PAD4-mediated histone citrullination weakens electrostatic interactions between histones and DNA, thereby promoting chromatin decondensation (Wang et al., 2004; Cuthbert et al., 2004; Christophorou et al., 2014; Leshner et al., 2012). This process is particularly evident under conditions requiring rapid genome-scale chromatin relaxation, as in NETosis, but it also occurs in transcriptionally active and signal-responsive chromatin domains. Histone citrullination may increase accessibility of transcription factors, coactivators, and chromatin remodelers to regulatory DNA elements, thereby enabling rapid changes in gene-expression programs.

A particularly important feature of histone citrullination is its crosstalk with arginine methylation. Arginine residues on histones can be modified by either methylation or citrullination, and these modifications often have distinct or opposing functional consequences (Wang et al., 2004; Cuthbert et al., 2004). Because citrullination removes the guanidinium group required for arginine methylation, PAD4 can antagonize methylation-dependent chromatin states. This provides a mechanism through which PAD4 reshapes the epigenetic landscape, not only by directly altering chromatin charge but also by competing with other regulatory modifications. The outcome may be activation or repression depending on the locus, the surrounding PTM environment, and the relevant transcriptional machinery.

### 3.2 Histone citrullination and transcriptional regulation

The relationship between PAD4 and transcription is context dependent and cannot be reduced to a simple activating or repressive role. In many situations, PAD4 facilitates transcription by opening chromatin and enabling access to regulatory DNA regions. In other contexts, PAD4 suppresses transcription through antagonism of activating methyl marks or through cooperation with repressive chromatin complexes (Wang et al., 2004; Cuthbert et al., 2004; Christophorou et al., 2014; Zhou et al., 2018; Wiese and Bannister, 2020). This apparent duality reflects the broader principle that chromatin-regulatory enzymes function within highly specific genomic and cellular contexts.

PAD4 can regulate gene expression through direct modification of histones at promoters and enhancers. Histone H3 citrullination has been linked to increased chromatin accessibility and locus-specific transcriptional activation (Cuthbert et al., 2004; Christophorou et al., 2014; Leshner et al., 2012). In pluripotent cells and differentiating immune cells, this can contribute to remodeling of lineage-specific transcriptional programs (Christophorou et al., 2014). In inflammatory settings, PAD4 may facilitate the expression of genes involved in cytokine production, leukocyte activation, and stress responses (Sun et al., 2017).

In addition to histones, PAD4 can influence transcription by modifying non-histone proteins involved in gene regulation. One of the most prominent examples is NF-κB p65, a central mediator of inflammatory gene expression. Citrullination of NF-κB p65 has been reported to enhance its nuclear localization and promote Toll-like receptor-induced expression of IL-1β and tumor necrosis factor-α (TNF-α) (Sun et al., 2017). These findings suggest that PAD4 integrates chromatin-level and transcription-factor-level control, thereby amplifying inflammatory transcriptional responses through multiple complementary mechanisms.

PAD4 may also interact functionally with hormone receptors, hypoxia-responsive pathways, and stress signaling systems. For example, PAD4-mediated histone citrullination has been linked to hypoxia-inducible factor (HIF)-dependent transcriptional responses in cancer and to broader chromatin remodeling programs associated with tumor adaptation (Wang et al., 2021). Such findings reinforce the concept that PAD4 is not restricted to neutrophil biology but also participates in broader signal-responsive transcriptional regulation across cell types.

### 3.3 PAD4 in chromatin decondensation and NETosis

**FIGURE 3:**
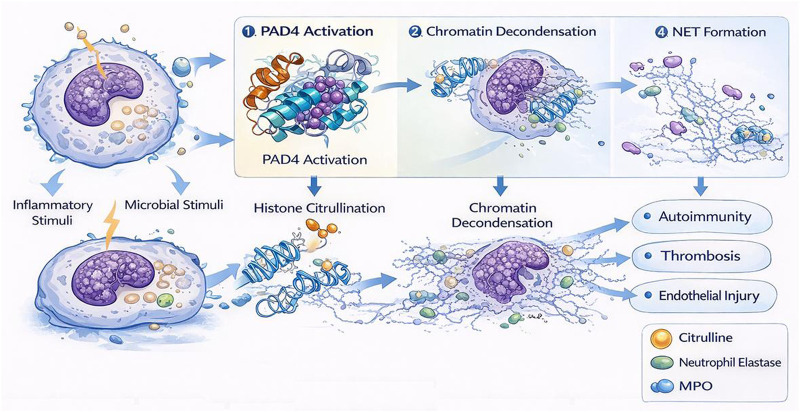
PAD4-dependent chromatin decondensation during neutrophil extracellular trap formation.

The most dramatic example of PAD4-driven chromatin remodeling occurs during NET formation. NETosis is a specialized form of neutrophil response characterized by large-scale chromatin decondensation, breakdown of nuclear organization, and extrusion of DNA decorated with histones, neutrophil elastase, myeloperoxidase, and other antimicrobial factors (Thiam et al., 2020a; Thiam et al., 2020b; Papayannopoulos, 2018; Clark et al., 2018; Liu et al., 2025; Fuchs et al., 2010; Tatsiy and McDonald, 2018; Li H. et al., 2025). PAD4 plays a central role in many forms of NETosis by catalyzing histone citrullination, which reduces histone-DNA affinity and promotes chromatin relaxation (Figure 3) (Thiam et al., 2020a; Lewis et al., 2015; Leshner et al., 2012; Clark et al., 2018).

NET formation can be triggered by microbial products, cytokines, activated platelets, immune complexes, cholesterol crystals, and sterile inflammatory stimuli (Thiam et al., 2020b; Papayannopoulos, 2018; Liu et al., 2025). Depending on the stimulus, NETosis may proceed through ROS-dependent or ROS-independent pathways, and the requirement for PAD4 may vary quantitatively across different contexts (Tatsiy and McDonald, 2018). Nevertheless, PAD4-mediated histone citrullination is one of the most consistently identified mechanistic drivers of chromatin decondensation during NET formation.

The process of NETosis involves coordinated cellular disassembly. Cytoskeletal remodeling, granule mobilization, nuclear swelling, and nuclear-envelope rupture precede chromatin release (Thiam et al., 2020a). PAD4-dependent citrullination contributes to the loss of higher-order chromatin compaction, enabling the chromatin mass to expand and ultimately be released into the extracellular space. The resulting NETs can trap bacteria, fungi, and other pathogens, increasing local antimicrobial concentration and reducing microbial dissemination (Li et al., 2010; Clark et al., 2018; Liu et al., 2025; McDonald et al., 2017; Alturki et al., 2025).

During NETosis, inflammatory or microbial stimuli induce PAD4 activation, histone citrullination, and large-scale chromatin decondensation. This is followed by nuclear swelling, disruption of nuclear architecture, and release of extracellular chromatin decorated with antimicrobial proteins such as neutrophil elastase and myeloperoxidase. NETs support host defense by trapping pathogens but can also promote autoimmunity, endothelial injury, and thrombosis when excessive or unresolved.

### 3.4 The protective and pathological roles of NETs

NETs are a paradigmatic example of how an evolutionarily protective immune mechanism can become pathological when excessive, unresolved, or misdirected. Under physiological conditions, NETs contribute to antimicrobial defense by immobilizing pathogens and exposing them to toxic enzymes and peptides (Clark et al., 2018; McDonald et al., 2017; Alturki et al., 2025). Experimental models have shown that PAD4 deficiency impairs NET formation and may reduce control of certain bacterial or fungal infections (Li et al., 2010; Liu et al., 2025; McDonald et al., 2017). These observations underscore the beneficial aspect of PAD4 activity in innate immunity.

When excessive or poorly cleared, NETs become pathogenic: extracellular chromatin, citrullinated histones, proteases, and DNA injure endothelium, activate platelets and macrophages, expose autoantigens, and provide a scaffold for fibrin deposition (Lood et al., 2016; Rosell et al., 2022; Curran et al., 2020; Kaplan, 2025; Fuchs et al., 2010; Döring et al., 2017; Jorch and Kubes, 2017). Thus, PAD4-dependent NETosis is protective during controlled antimicrobial defense but injurious when it persists or occurs in sterile inflammatory and thrombotic settings.

### 3.5 PAD4 in inflammatory signaling beyond NETosis

Although PAD4 is often discussed primarily in relation to NET formation, its role in inflammation extends beyond NETosis. PAD4 can modulate inflammatory gene expression, cytokine production, and cellular activation states through both chromatin-dependent and chromatin-independent mechanisms (Sun et al., 2017; Santos B et al., 2026; Nakashima et al., 1999; Knight et al., 2013). Elevation of intracellular calcium during inflammatory stress activates PAD4, while the local redox environment may further shape enzyme function by modifying catalytic residues or interacting regulatory pathways (Santos B et al., 2026; Nakashima et al., 1999; Knight et al., 2013).

Through modification of transcriptional regulators such as NF-κB, PAD4 can amplify the production of TNF-α, interleukin-1β (IL-1β), and other inflammatory mediators (Sun et al., 2017). This may reinforce positive-feedback loops in innate immune signaling and promote persistent tissue inflammation. In macrophages, dendritic cells, and other myeloid populations, PAD4-dependent or PAD4-associated pathways may influence cytokine output, antigen presentation, and crosstalk with adaptive immunity. Even when PAD4 is not the sole driver of these processes, it may function as an amplifier that enhances inflammatory intensity and duration.

PAD4 has also been linked to cellular differentiation, apoptosis, and epithelial-mesenchymal transition (EMT), particularly in cancer-related contexts (Wu et al., 2023; Shi et al., 2020; Wang et al., 2022; Wang et al., 2021; Cristinziano et al., 2022; Zhu et al., 2022; Chang and Han, 2006; Stadler et al., 2013; Li J. et al., 2025). These broader roles likely reflect the convergence of epigenetic regulation, transcriptional reprogramming, and structural protein modification. Thus, PAD4 should not be viewed solely as a neutrophil enzyme but rather as a multifunctional regulator of inflammatory and stress-responsive cellular states.

### 3.6 Why these mechanisms matter pharmacologically

The molecular properties described above make PAD4 particularly attractive from a pharmacological perspective. First, PAD4 occupies an upstream mechanistic position in several disease-relevant pathways, including histone citrullination, NETosis, inflammatory transcription, and immunothrombosis. Targeting PAD4 therefore offers the possibility of modulating multiple downstream pathological processes simultaneously. Second, the enzyme possesses a structurally defined catalytic pocket and well-characterized conformational states, enabling rational drug design (Aiken et al., 2025; Arita et al., 2004; Knuckley et al., 2010; Zhou et al., 2018; Dakin et al., 2025). Third, the pathological effects of PAD4 appear to be especially prominent under conditions of exaggerated inflammation, which raises the possibility of a therapeutic window in diseases driven by dysregulated rather than basal PAD4 activity.

At the same time, the same biology that makes PAD4 attractive also creates translational complexity. Because PAD4 contributes to host defense and to context-dependent gene regulation, indiscriminate inhibition may carry risks related to infection, impaired immune adaptation, or unexpected tissue effects. PAD4’s upstream position in chromatin regulation, NETosis, and inflammatory transcription makes it an attractive drug target, but its physiological roles demand selective rather than pan-suppressive pharmacological strategies.

## 4 PAD4 in human disease

To avoid repetition, the disease discussion below is organized around shared PAD4-dependent mechanisms rather than as isolated disease narratives. Across pathological settings, PAD4 contributes mainly through five overlapping axes: citrullinated neoepitope generation, chromatin and transcriptional regulation, NET-dependent tissue injury, extracellular matrix remodeling, and immunothrombosis. Table 2 summarizes the disease-specific manifestations of these axes, whereas the text highlights only the most distinctive evidence and therapeutic implications.

**TABLE 2:** Pathogenic roles of PAD4 across major disease contexts.

Disease	Main PAD4-associated mechanism	Major pathogenic consequence	Therapeutic implication
Rheumatoid arthritis	Citrullinated neoantigen generation; NET-derived autoantigen exposure; inflammatory amplification in synovium	Anti-citrullinated protein antibodies formation, immune-complex signaling, synovial inflammation, tissue destruction	PAD4 inhibition may reduce citrullination, NETosis, and inflammatory amplification (Suzuki et al., 2003; Curran et al., 2020; Chen et al., 2024)
Systemic lupus erythematosus	PAD4-dependent NET formation; exposure of DNA-histone complexes; interferogenic autoantigen release	Type I interferon activation, autoantibody production, vascular and renal injury	PAD4 inhibition may suppress NET-driven immune activation and tissue damage (Lood et al., 2016; Kaplan, 2025; Seidlberger et al., 2026)
Multiple sclerosis/CNS demyelination	Citrullination of myelin basic protein; possible contribution to neuroinflammation and NET-associated immune activation	Myelin destabilization, inflammatory lesion progression, tissue damage	PAD-targeted approaches may attenuate demyelination, although the PAD4-specific contribution remains uncertain (Moscarello et al., 2002; Martinsen and Kursula, 2022; Yan et al., 2016; Geng et al., 2023)
Cancer	Histone citrullination, transcriptional reprogramming, epithelial-mesenchymal transition, extracellular matrix remodeling, metastatic niche formation	Tumor progression, invasion, metastasis, microenvironmental adaptation	PAD4 inhibition may impair tumor plasticity and metastatic progression in selected cancers (Shi et al., 2020; Zhu et al., 2022; Stadler et al., 2013)
Cancer-associated thrombosis	NET formation and citrullinated histone-driven coagulation	Platelet adhesion, fibrin deposition, hypercoagulability, thrombotic complications	PAD4 inhibition may simultaneously reduce thromboinflammation and thrombosis (Rosell et al., 2022; Fuchs et al., 2010; Cristinziano et al., 2022; Li et al., 2025b)
Atherosclerosis	NET-mediated endothelial dysfunction and macrophage activation	Plaque progression, chronic vascular inflammation, lesion instability	PAD4 inhibition may attenuate vascular inflammation and plaque development (Knight et al., 2014; Warnatsch et al., 2015; Franck et al., 2018)
Acute coronary syndromes and arterial thrombosis	NET-dependent immunothrombosis at sites of plaque rupture or erosion	Coronary thrombosis, infarct extension, endothelial injury	PAD4 inhibition may reduce thrombus burden and ischemic injury (Franck et al., 2018; Mangold et al., 2015)
Infection and sepsis	Protective NETosis during infection; pathological NET excess in severe systemic inflammation	Antimicrobial defense on one hand; endothelial damage, microvascular thrombosis, organ failure on the other	Therapeutic utility depends on timing and disease context; excessive inhibition may compromise host defense (Li et al., 2010; McDonald et al., 2017; McDonald et al., 2012; Bonilha et al., 2025)

### 4.1 PAD4 in autoimmune disease

Autoimmune disorders represent one of the best established clinical settings in which PAD4-mediated citrullination is pathogenic. In these diseases, citrullination can convert endogenous proteins into neoantigens, facilitate epitope spreading, and sustain chronic inflammatory circuits. PAD4 is particularly relevant in rheumatoid arthritis and systemic lupus erythematosus, where immune dysregulation, NET formation, and exposure of nuclear antigens converge to amplify disease progression (Suzuki et al., 2003; Lood et al., 2016; Curran et al., 2020; Kaplan, 2025).

#### 4.1.1 Rheumatoid arthritis

RA provides the clearest example of PAD4-driven autoantigen generation. In the inflamed joint, PAD4 derived from neutrophils, macrophages, and fibroblast-like synoviocytes citrullinates proteins such as fibrinogen, vimentin, alpha-enolase, and type II collagen, generating neoepitopes recognized by anti-citrullinated protein antibodys (ACPAs) (Suzuki et al., 2003; Curran et al., 2020; Chen et al., 2024; Kimura et al., 2023; Thomas et al., 2024; Pollmann et al., 2012; Zhang G. et al., 2024; Cappelli et al., 2024; Salemme et al., 2019; Baños-Hernández et al., 2017). ACPA-containing immune complexes can activate complement and Fc-receptor-bearing innate immune cells, thereby linking citrullination directly to synovial inflammation.

PAD4 also amplifies RA through NETosis. NETs in synovial fluid and joint tissue contain citrullinated histones and other autoantigenic proteins that stimulate macrophages and fibroblast-like synoviocytes to produce TNF-alpha, IL-6, IL-1beta, and related mediators (Curran et al., 2020; Chen et al., 2024; Baños-Hernández et al., 2017). Thus, the central PAD4 axis in RA is not simply antigen creation but the coupling of neoepitope exposure with a self-sustaining inflammatory microenvironment.

Genetic evidence further supports a role for PAD4 in RA susceptibility. Functional haplotypes and polymorphisms within PADI4 have been associated with RA in several populations, particularly in Asian cohorts (Kimura et al., 2023; Zhang G. et al., 2024). These variants may increase mRNA stability or protein expression, thereby enhancing citrullination capacity. However, the strength and reproducibility of the genetic association differ across ethnic groups, suggesting that PADI4 acts within a broader gene-environment framework rather than as a universal determinant of disease risk.

Beyond its enzymatic role, PAD4 also functions as an autoimmune target. Anti-PAD4 autoantibodies have been detected in subsets of RA patients and have been associated with more severe disease phenotypes in some studies (Salemme et al., 2019). Although their incremental diagnostic value beyond ACPA and rheumatoid factor is limited, anti-PAD4 antibodies may prove useful for disease stratification, prognosis, or treatment response prediction. Overall, the evidence strongly supports PAD4 as a multifunctional pathogenic mediator in RA, acting through autoantigen generation, inflammatory amplification, and NET-dependent immune activation.

#### 4.1.2 Systemic lupus erythematosus

In systemic lupus erythematosus (SLE), PAD4 is most relevant through NET-dependent exposure of immunostimulatory nuclear material. Neutrophils from patients with SLE show enhanced NETosis, and their NETs are enriched in citrullinated histones, DNA, and oxidized mitochondrial components that activate plasmacytoid dendritic cells, promote type I interferon production, and stimulate autoreactive B cells (Lood et al., 2016; Geng et al., 2023; Kaplan, 2025; Seidlberger et al., 2026; Kaan et al., 2024; Rohrbach et al., 2012; Villanueva et al., 2011; Massarenti et al., 2019).

The pathogenic impact of PAD4 in SLE is intensified when NET clearance is defective. Impaired DNase activity, complement defects, or inefficient phagocytic processing prolong exposure to PAD4-generated autoantigens and can sustain the cycle of extracellular chromatin exposure, interferon signaling, immune-complex formation, and tissue injury (Kaan et al., 2024; Massarenti et al., 2019).

Genetic studies have linked PADI4 polymorphisms to lupus susceptibility and, in some cohorts, to lupus nephritis (Massarenti et al., 2019). Although these associations require further clarification across populations, they reinforce the concept that PAD4 may contribute to both systemic autoimmunity and organ-specific pathology. Pharmacological PAD inhibition has shown benefit in murine lupus models, with reductions in NET formation, vascular injury, and immune activation (Knight et al., 2013). These findings support PAD4 as more than a biomarker of lupus-associated neutrophil activation; they argue for a mechanistically meaningful therapeutic target.

#### 4.1.3 Potential involvement in multiple sclerosis and central nervous system demyelinating disease

PAD-mediated citrullination has long been implicated in central nervous system demyelination, although PAD4-specific evidence is weaker than that for PAD2 in multiple sclerosis (MS) (Moscarello et al., 2002; Martinsen and Kursula, 2022; Yan et al., 2016; Geng et al., 2023). Citrullination of arginine-rich myelin basic protein reduces its positive charge, weakens interactions with negatively charged myelin lipids, and may increase susceptibility to myelin disassembly and proteolysis (Martinsen and Kursula, 2022; Yan et al., 2016).

PAD4 has been detected in inflammatory infiltrates, macrophages, microglia, and glial cells under neuroinflammatory conditions, suggesting that it may contribute indirectly through local immune activation as well as directly through substrate citrullination (Moscarello et al., 2002; Geng et al., 2023). At present, PAD4 should therefore be presented as a plausible but incompletely defined contributor to central nervous system (CNS) demyelinating disease.

### 4.2 PAD4 in cancer

Aberrant PAD4 expression or activity has been reported in multiple malignancies, including breast, lung, colorectal, gastric, hepatic, ovarian, and endometrial cancers (Wu et al., 2023; Wang et al., 2022; Wang et al., 2021; Zhu et al., 2022; Chang and Han, 2006; Stadler et al., 2013). Current evidence indicates that PAD4 can influence tumor biology through several interacting mechanisms, including epigenetic regulation within tumor cells, promotion of tumor-cell plasticity, extracellular matrix remodeling, facilitation of metastatic niche formation, and NET-associated thrombosis (Shi et al., 2020; Wang et al., 2022; Tomita et al., 2025; Zhu et al., 2022).

PAD4 promotes cancer through tumor-intrinsic epigenetic reprogramming, stromal remodeling, NET-dependent thrombosis, and immunosuppression via (tumor-associated macrophages) TAMs; its inhibition may thus offer multi-faceted therapeutic benefits in selected cancers.

#### 4.2.1 PAD4 overexpression and tumor-cell biology

In cancer, PAD4 can influence malignant behavior through chromatin remodeling, non-histone citrullination, and tumor-microenvironmental regulation. Experimental studies indicate that PAD4 inhibition or knockdown can reduce proliferation, induce apoptosis, perturb cell-cycle progression, and interfere with programs linked to hypoxia, TGF-beta signaling, EMT, and tumor vascularization (Wang et al., 2022; Wang et al., 2021; Cristinziano et al., 2022; Zhu et al., 2022; Chang and Han, 2006; Stadler et al., 2013). Recent evidence also shows that macrophage PAD4 can restrain MHC class II machinery through STAT1 citrullination, suggesting that PAD4 may suppress antitumor immunity in selected tumor microenvironments (TME) (Pitter et al., 2024).

#### 4.2.2 PAD4 in invasion, extracellular matrix remodeling, and metastasis

PAD4 may further support invasion and metastasis by acting outside the nucleus. Citrullination of extracellular matrix proteins such as collagen and fibronectin can alter matrix integrity and proteolytic susceptibility, while PAD4-dependent extracellular chromatin networks may promote tumor-cell adhesion, stromal interactions, colonization, and immune evasion (Shi et al., 2020; Wang et al., 2022; Tomita et al., 2025; Li J. et al., 2025). Thus, PAD4 is better viewed as a context-dependent facilitator of metastatic competence than as a universal metastasis driver.

#### 4.2.3 PAD4, NETs, and cancer-associated thrombosis

Cancer-associated thrombosis illustrates the shared NET-immunothrombosis axis discussed above. PAD4-dependent NETs provide DNA-histone scaffolds for platelet adhesion, fibrin deposition, thrombin generation, and vascular injury, and elevated circulating NET markers or citrullinated histones have been linked to thrombotic complications in cancer (Rosell et al., 2022; Fuchs et al., 2010; Döring et al., 2017; Cristinziano et al., 2022). This dual involvement in tumor-supportive inflammation and thrombosis makes PAD4 an attractive, but still largely preclinical, oncology target.

At present, however, most mechanistic evidence for PAD4 in cancer remains preclinical. The biological importance of PAD4 likely differs across tumor types, stages, and microenvironmental states. Accordingly, while PAD4 is a promising target in oncology, future work must better define when its inhibition is most likely to yield meaningful therapeutic benefit.

Beyond its roles in tumor-cell-intrinsic biology and thrombosis, PAD4 also shapes the tumor immune microenvironment through effects on tumor-associated macrophages (TAMs). PAD4 was found to be among the most highly expressed post-translational modification enzymes in TAMs from both mouse and human tumors, and its expression negatively correlated with clinical response to immune checkpoint blockade (Pitter et al., 2024). Mechanistically, PAD4 citrullinates signal transducer and activator of transcription 1 (STAT1) at arginine 121 in macrophages, thereby promoting the protein inhibitor of activated STAT1 (PIAS1) interaction and restraining STAT1 transcriptional activity. This leads to reduced expression of MHC class II machinery, impaired antigen presentation, and diminished T cell-mediated antitumor immunity. Genetic or pharmacological inhibition of PAD4 in macrophages reversed these effects, increasing MHC class II expression, enhancing T cell effector function, and suppressing tumor progression in multiple mouse models. These findings expand the role of PAD4 in cancer beyond neutrophil-driven NETosis and highlight its function as an immune-restrictive node in the tumor microenvironment. Consequently, PAD4 inhibition may offer a dual benefit in cancer—simultaneously suppressing tumor-cell-supportive citrullination and relieving PAD4-mediated immune evasion in TAMs.

### 4.3 PAD4 in cardiovascular disease

A growing body of evidence implicates PAD4 in cardiovascular pathology, particularly in atherosclerosis, acute coronary syndromes (ACS), arterial thrombosis, and ischemia-reperfusion injury (Knight et al., 2014; Döring et al., 2017; Mangold et al., 2015; Mand et al., 2025). In these settings, PAD4 acts largely through immunothrombotic mechanisms centered on NET formation, endothelial damage, and amplification of vascular inflammation.

#### 4.3.1 PAD4 and atherosclerosis

Atherosclerosis is a chronic inflammatory disease in which PAD4-dependent NETosis contributes to vascular injury and plaque evolution (Warnatsch et al., 2015; Franck et al., 2018; Mangold et al., 2015; Silvestre-Roig et al., 2019; Lin et al., 2025; Kindberg et al., 2025). NET-derived histones, proteases, and DNA can damage endothelium, increase oxidative stress, stimulate macrophage cytokine production, and act as damage-associated molecular patterns (DAMPs) that sustain lesion inflammation (Franck et al., 2018; Mangold et al., 2015; Silvestre-Roig et al., 2019; Lin et al., 2025).

Consistent with this mechanism, PAD4 deficiency or pharmacological PAD inhibition has reduced NET formation, vascular inflammation, and lesion burden in some murine models (Knight et al., 2014; Mangold et al., 2015; Lin et al., 2025). However, PAD4 effects may vary by lesion stage, cellular compartment, and inflammatory context, so its role in atherogenesis should be interpreted as important but not uniform across all models (Kindberg et al., 2025).

#### 4.3.2 PAD4 in acute coronary syndromes and thrombosis

In acute coronary syndromes (ACS), PAD4 contributes mainly through rapid NET-dependent immunothrombosis after plaque rupture, plaque erosion, or endothelial injury (Franck et al., 2018; Mangold et al., 2015; Kindberg et al., 2025). NETs create a procoagulant surface for platelet aggregation, fibrin accumulation, and thrombin generation, while tissue factor-bearing NETs have been identified in culprit coronary lesions (Mangold et al., 2015).

Circulating NET markers, including cell-free DNA and citrullinated histones, correlate with adverse cardiovascular outcomes and infarct burden, and preclinical PAD4 inhibition can reduce thrombus formation, infarct size, and ischemia-associated cardiac injury (Knight et al., 2014; Franck et al., 2018; Mangold et al., 2015; Mand et al., 2025; Kindberg et al., 2025). Clinical translation will require balancing antithrombotic benefit against possible effects on host defense.

### 4.4 PAD4 in host defense, infection, and sepsis

In infection, PAD4 has a dual role that should be stated once and applied consistently: controlled PAD4-dependent NETosis supports antimicrobial defense, whereas excessive or persistent activation promotes endothelial injury, coagulopathy, and organ dysfunction (Li et al., 2010; Liu et al., 2025; McDonald et al., 2017; Bonilha et al., 2025). During host defense, histone citrullination enables chromatin release and formation of NETs enriched in neutrophil elastase, myeloperoxidase, defensins, and other antimicrobial molecules.

In sepsis, the same pathway can become maladaptive. Excessive NETs release cytotoxic histones and DNA, promote platelet-NET interactions, accelerate microvascular thrombosis, and amplify inflammatory signaling, including NF-κB-related cytokine responses (Sun et al., 2017; McDonald et al., 2017; McDonald et al., 2012; Bonilha et al., 2025). PAD4 inhibition may therefore be useful only when immunothrombotic injury outweighs antimicrobial benefit, making timing, dose, and patient selection essential.

## 5 Therapeutic targeting of PAD4

The growing understanding of PAD4 biology has made it an increasingly attractive therapeutic target in inflammation, autoimmunity, thrombosis, cancer, and infection-associated tissue injury. Several features support its druggability. PAD4 sits upstream of multiple disease-relevant processes, including histone citrullination, chromatin remodeling, inflammatory gene regulation, NET formation, and immunothrombosis. It also contains a structurally defined catalytic pocket centered on a reactive cysteine residue, which provides a clear starting point for inhibitor design (Arita et al., 2004; Luo et al., 2006a; Causey et al., 2011). Finally, substantial preclinical evidence indicates that suppression of PAD4 activity can reduce pathological citrullination and modify disease phenotypes in several experimental systems (Lewis et al., 2015; Knight et al., 2014; Knight et al., 2013; Gajendran et al., 2023).

**FIGURE 4:**
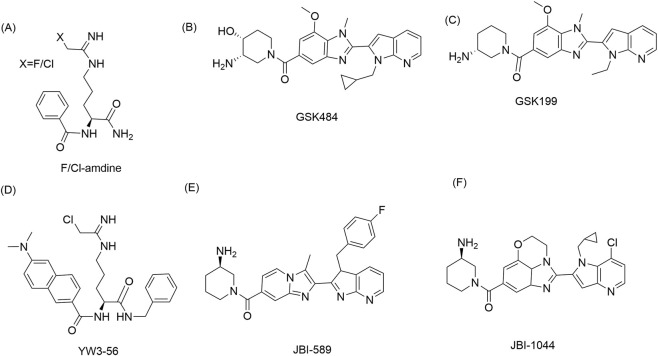
Representative chemical scaffolds of PAD4 inhibitors. **(A)** F/Cl‐amidine, **(B)** GSK484, **(C)** GSK199, **(D)** YW3‐56, **(E)** JBI‐589, **(F)** JBI‐1044.

Although PAD4 has emerged as a compelling therapeutic target, the clinical translation of PAD4 inhibitors remains challenging. Classical amidine-based compounds such as Cl-amidine were invaluable as tool molecules but are limited by irreversible binding and modest isoform selectivity. More selective reversible inhibitors, including GSK484 and GSK199, have enabled cleaner interrogation of PAD4 biology and strengthened the case that PAD4 is a tractable target in preclinical systems (Figure 4).

The recent discovery that GAGs activate PAD4 by enhancing calcium affinity also holds therapeutic implications (Bereta et al., 2025). On the one hand, GAG-rich inflammatory sites may promote pathological PAD4 activity even at otherwise sub-activatory extracellular calcium concentrations; on the other hand, the GAG-binding interface—which involves positively charged surface patches rather than a classical binding pocket—may offer opportunities for novel inhibitor or biologic strategies that disrupt GAG–PAD4 interactions without directly targeting the catalytic center.

At the same time, therapeutic targeting of PAD4 requires careful consideration of biological context. PAD4 participates in host defense, and its role is not uniformly pathogenic across all tissues or stimuli. Therefore, the goal of drug development is not simply maximal enzyme suppression, but rather the design of inhibitors with sufficient selectivity, pharmacokinetic suitability, and therapeutic precision to attenuate disease-driving PAD4 activity while minimizing interference with physiological immune function. The current PAD4-targeting landscape includes irreversible covalent inhibitors, reversible conformation-selective inhibitors, orally available translational candidates, multifunctional compounds, and emerging modalities such as antibodies, cyclic peptides, and allosteric ligands.

Illustrated are representative PAD4-targeted small molecules, including early irreversible inhibitors and later reversible or selective compounds. These chemical classes highlight the evolution of PAD4 drug discovery from catalytic cysteine-directed covalent inhibition toward conformation-sensitive and more selective pharmacological strategies.

### 5.1 Why PAD4 is a tractable pharmacological target

PAD4 satisfies many of the classical criteria of a tractable enzyme target. Its catalytic mechanism is well characterized, its structural organization has been resolved in detail, and its active site includes chemically targetable residues (Arita et al., 2004; Luo et al., 2006a). In addition, PAD4-mediated histone citrullination and NET formation provide measurable pharmacodynamic outputs that can be assessed experimentally, enabling mechanistic evaluation of target engagement (Thiam et al., 2020a; Lewis et al., 2015; Leshner et al., 2012; Clark et al., 2018). This is especially important in translational pharmacology, where the ability to link drug exposure to pathway suppression is essential.

The disease relevance of PAD4 is also unusually broad. Unlike targets that are confined to a narrow disease niche, PAD4 sits at the convergence of several major pathological programs: chronic inflammation, loss of immune tolerance, endothelial injury, thrombosis, tumor progression, and dysregulated host defense. This gives PAD4 inhibitors potential utility across multiple indications. However, it also raises a challenge: because PAD4 participates in distinct disease mechanisms in different tissues, the optimal inhibitor profile may not be the same for autoimmune disease, cardiovascular thrombosis, cancer, and sepsis. Some settings may benefit from sustained systemic inhibition, whereas others may require short-term or tissue-restricted modulation.

**FIGURE 5:**
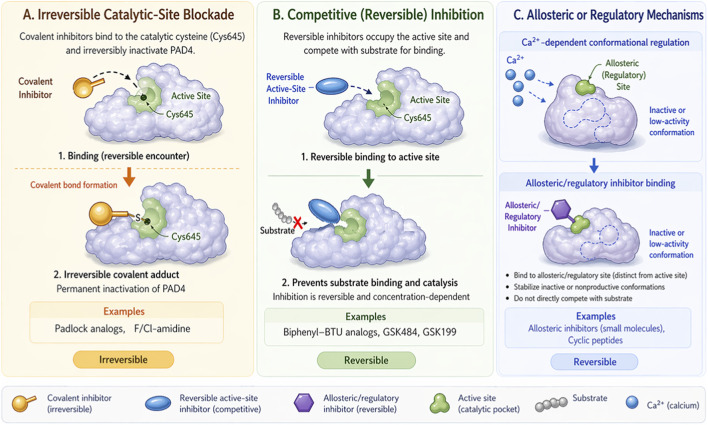
Mechanisms of PAD4 inhibition. **(A)** Irreversible catalytic‐site blockade: covalent inhibitors bind Cys645, permanently inactivating the enzyme. **(B)** Competitive reversible inhibition: reversible inhibitors occupy the active site, blocking substrate binding. **(C)** Allosteric or regulatory inhibition: inhibitors or calcium bind allosteric sites, inducing inactive conformations.

From a medicinal chemistry perspective, the development of PAD4 inhibitors has also revealed an important conceptual evolution (Figure 5). Early compounds were designed primarily to exploit the catalytic cysteine through irreversible covalent chemistry (Causey et al., 2011; Luo et al., 2006b). Later work demonstrated that selective and reversible inhibition could be achieved by stabilizing inactive or calcium-deficient conformations of PAD4 (Lewis et al., 2015; Aiken et al., 2025). Most recently, allosteric and nontraditional modalities have expanded the design space beyond classical active-site blockade (Dakin et al., 2025; Zhou et al., 2024; Bertran et al., 2024). This progression mirrors the maturation of PAD4 drug discovery from target validation to mechanism-informed optimization (Table 3).

**TABLE 3:** Representative PAD4 inhibitors and emerging therapeutic modalities.

Inhibitor/Modality	Type	Mechanism of action	Key features	Main applications or models	Main limitations
Cl-amidine (Luo et al., 2006a; Chumanevich et al., 2011)	Irreversible small molecule	Covalent modification of catalytic cysteine	Foundational PAD inhibitor; important proof-of-concept tool	Inflammation, lupus, NETosis, and mechanistic tool studies	Limited isoform selectivity; suboptimal clinical drug-like properties
F-amidine (Luo et al., 2006b)	Irreversible small molecule	Covalent catalytic-site inhibition	Early validation compound for PAD inhibition	Early *in vitro* and *in vivo* PAD inhibition studies	Limited selectivity and translational suitability
o-F-amidine/o-Cl-amidine (Causey et al., 2011)	Second-generation irreversible analogs	Covalent inhibition with scaffold optimization	Improved potency/selectivity relative to first-generation amidines	Mechanistic and preclinical studies	Still not ideal as advanced clinical candidates
GSK484 [16,24]	Reversible small molecule	Binds inactive/low-calcium PAD4 conformation	Selective PAD4 probe; suppresses histone citrullination and NET formation	NETosis and inflammatory signaling studies	Primarily a tool compound
GSK199 [24]	Reversible small molecule	Non-covalent inhibition of calcium-deficient PAD4	Important structure-guided scaffold with PAD4 selectivity	Medicinal chemistry benchmark compound	Surpassed by later compounds with better PK and *in vivo* performance
YW3-56 [97]	Multifunctional inhibitor	PAD4 inhibition with additional effects on tumor-related signaling	Illustrates polypharmacological or context-oriented strategy	Cancer and leukemia-related models	Complex mechanism; translational role not fully defined
JBI-589 [92]	Orally available reversible inhibitor	Selective non-covalent PAD4 inhibition	Strong PAD4 selectivity; inhibits NET formation; translationally promising	Mouse arthritis and neutrophil-based models	Clinical-stage validation still limited
JBI-1044 [24]	Advanced translational candidate	Selective PAD4 inhibition	Reported progress toward candidate-quality development	Fibrosis, colitis, and cancer-related preclinical models	Public clinical data remain limited
Anti-PAD4 antibodies (Zhou et al., 2024)	Biologic modality	Regulatory or inhibitory antibody-based modulation	Potential for non-classical selectivity and pathway control	Emerging mechanistic and therapeutic studies	Early-stage modality
Cyclic peptides (Bertran et al., 2024)	Peptide-based modulators	Conformation-sensitive modulation of PAD4	Useful for probing regulation and selectivity	Mechanistic studies	Delivery and pharmacokinetic challenges
Allosteric ligands (Dakin et al., 2025)	Emerging small-molecule strategy	Target calcium-dependent allosteric pocket	Expands design space beyond active site	Next-generation PAD inhibitor discovery	Early in development; selectivity and translation still under study

The discovery that GAGs can allosterically enhance PAD4 calcium sensitivity has important therapeutic implications. PAD4 inhibitors may need to retain efficacy not only against purified calcium-activated PAD4, but also against GAG-stabilized higher-order PAD4 assemblies that may occur in inflamed extracellular matrices and synovial fluid (Bereta et al., 2025). Therefore, future inhibitor evaluation should include disease-mimetic biochemical conditions, rather than relying solely on standard high-calcium assays (Bereta et al., 2025).

PAD4 activity may be inhibited through several complementary strategies, including irreversible catalytic-site blockade, reversible binding to inactive or calcium-deficient conformations, and emerging allosteric or regulatory mechanisms. These approaches differ in selectivity, pharmacological control, and translational potential, and together define the current therapeutic landscape of PAD4 targeting.

### 5.2 Irreversible inhibitors

#### 5.2.1 Cl-amidine

**FIGURE 6:**
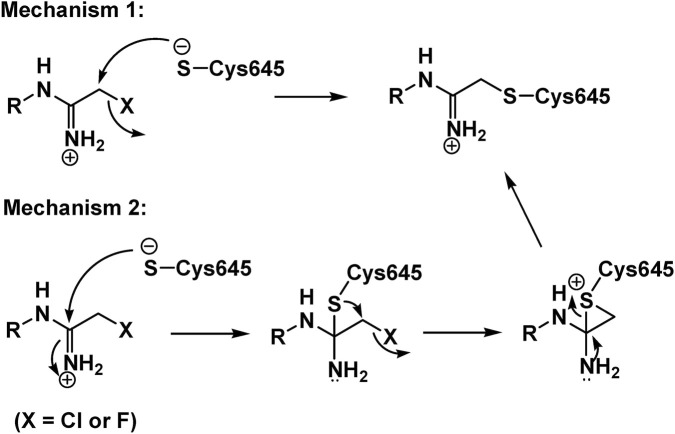
Two potential mechanisms of PAD4 inactivation.

Cl-amidine is among the earliest and most influential PAD inhibitors and played a foundational role in establishing PAD4 as a druggable therapeutic target (Luo et al., 2006a; Chumanevich et al., 2011). As a haloacetamidine-based compound, Cl-amidine irreversibly inactivates PAD enzymes through covalent modification of the catalytic cysteine residue (Figure 6). This mechanism produces durable enzyme blockade and made Cl-amidine especially useful in early proof-of-concept studies designed to test the pathological relevance of PAD activity.

This schematic illustrates two proposed mechanisms by which haloacetamidine-based PAD4 inhibitors inactivate PAD4 through covalent modification of the catalytic residue Cys645. In both pathways, the thiolate of Cys645 attacks the inhibitor, ultimately forming a stable PAD4-inhibitor adduct and blocking catalytic activity (Chumanevich et al., 2011).

Functionally, Cl-amidine suppresses histone citrullination, reduces NET formation, and attenuates disease-associated phenotypes in a range of inflammatory and autoimmune models (Knight et al., 2014; Knight et al., 2013; Chumanevich et al., 2011). In NET-driven settings, it helped establish that pharmacological disruption of PAD activity can impair pathological chromatin decondensation and NETosis. Cl-amidine therefore served as a key bridge between mechanistic PAD biology and experimentally testable therapeutic hypotheses.

Despite its historical importance, Cl-amidine also illustrates the limitations of first-generation PAD inhibitors. Its selectivity across PAD isoforms is limited, raising the possibility of off-target inhibition of related PAD family members (Aiken et al., 2025; Causey et al., 2011; Luo et al., 2006b). In addition, irreversible covalent chemistry may increase the risk of nonspecific protein reactivity and long-term tolerability concerns, particularly in chronic diseases requiring sustained treatment. The compound also lacks the pharmacokinetic refinement expected of a modern clinical candidate. Accordingly, Cl-amidine is best regarded as a landmark pharmacological tool compound rather than an optimized therapeutic agent.

#### 5.2.2 F-amidine and second-generation haloacetamidines

F-amidine belongs to the same early generation of amidine-based PAD inhibitors and likewise acts through irreversible covalent inactivation of the catalytic cysteine (Luo et al., 2006a; Causey et al., 2011). Like Cl-amidine, it provided important early evidence that pharmacological blockade of PAD enzymes can reduce pathological citrullination *in vitro* and *in vivo*. In this sense, F-amidine contributed to the first wave of chemical validation of PAD4 as a therapeutic target.

However, F-amidine shares many of the same limitations as Cl-amidine, including limited isoform selectivity and a pharmacological profile that is not ideal for clinical translation (Causey et al., 2011). Medicinal chemistry efforts subsequently generated improved derivatives such as o-F-amidine and o-Cl-amidine, designed to increase potency and selectivity (Causey et al., 2011). These second-generation analogs represented meaningful advances over the original haloacetamidine scaffold, but the field ultimately shifted toward reversible and more PAD4-focused chemotypes.

### 5.3 Reversible inhibitors

The development of reversible inhibitors marked a major conceptual and practical advance in PAD4 pharmacology. Reversible compounds offer the possibility of improved selectivity, reduced risk of permanent off-target protein modification, and better control over the duration and context of target suppression. They also exploit the conformational dynamics of PAD4, particularly its calcium-dependent activation states, rather than relying solely on active-site covalent chemistry.

#### 5.3.1 GSK484

GSK484 was a landmark compound because it demonstrated that selective and reversible inhibition of PAD4 could be achieved without covalent modification of the catalytic cysteine (Lewis et al., 2015). Rather than simply occupying the active site, GSK484 preferentially binds a low-calcium, inactive conformation of PAD4 and thereby prevents its transition to an active catalytic state. This mechanism represented a major advance by showing that PAD4 could be targeted through conformational control.

Biologically, GSK484 inhibits histone citrullination and suppresses NET formation in both human and murine neutrophils (Lewis et al., 2015). As a result, it quickly became one of the most widely used tool compounds for studying PAD4-dependent NETosis and inflammatory signaling. Relative to the early haloacetamidine inhibitors, GSK484 offered improved PAD4 selectivity and a cleaner framework for mechanistic studies. It helped establish the principle that selective PAD4 inhibition could be achieved by leveraging enzyme conformational states rather than nonspecific covalent reactivity.

Nevertheless, GSK484 is better viewed as a benchmark probe than a fully optimized therapeutic candidate. Although highly valuable experimentally, later drug-discovery efforts have sought to retain its mechanistic advantages while further improving potency, pharmacokinetics, and *in vivo* performance (Aiken et al., 2025; Gajendran et al., 2023). Even so, its importance in the PAD4 field cannot be overstated: GSK484 shifted the intellectual center of PAD4 medicinal chemistry toward reversible and conformation-sensitive inhibition.

#### 5.3.2 GSK199

GSK199 was the first submicromolar non-covalent PAD4 inhibitor described from a distinct medicinal chemistry scaffold and remains one of the most influential compounds in the PAD4 inhibitor literature (Aiken et al., 2025). Structural analyses indicated that GSK199 binds calcium-deficient PAD4 and stabilizes a conformation favorable for inhibitor recognition. Interactions with residues such as Phe634, which are not fully conserved across PAD isoforms, appear to contribute to its PAD4 selectivity (Aiken et al., 2025).

GSK199 became a reference scaffold for subsequent PAD4 discovery programs in both academic and industrial settings. Many later inhibitor series either evolved directly from the GSK199 chemotype or adopted related design principles involving optimization of the heteroaromatic core and flanking substituents (Aiken et al., 2025). In this sense, GSK199 was important not only as an inhibitor but also as a platform that defined the modern medicinal chemistry logic of PAD4-targeted small molecules.

Like GSK484, however, GSK199 was eventually surpassed by more advanced compounds with better potency, exposure, and *in vivo* efficacy (Aiken et al., 2025; Gajendran et al., 2023). Its enduring significance lies in how it demonstrated that highly selective PAD4 inhibition could be built from rational structure-guided design, thereby supporting the transition from tool compounds to candidate-quality molecules.

### 5.4 Tumor-targeted and multifunctional inhibitors

The complex biology of PAD4 in cancer and inflammation has stimulated interest in multifunctional inhibitors that do more than simply block catalytic activity. Such compounds are designed either to target PAD4 within specific disease contexts or to modulate additional signaling pathways that cooperate with PAD4-dependent pathogenesis.

YW3-56 is a representative example of this strategy. It has been explored as a multifunctional PAD4-directed compound in cancer-related models and illustrates interest in integrating PAD4 inhibition with broader regulation of malignant cell state (Zhu et al., 2025). The broader implication of this type of compound is that PAD4 inhibition may prove more effective when combined with modulation of downstream stress-response, metabolic, or transcriptional programs.

The appeal of such multifunctional agents is especially strong in oncology, where pathway redundancy and adaptive resistance often limit the efficacy of highly selective single-target therapies. A dual-function or context-sensitive PAD4 inhibitor could, in principle, attenuate both upstream citrullination and downstream disease-promoting signaling. However, such strategies also complicate mechanism-of-action analysis and may introduce additional safety considerations. For these reasons, multifunctional PAD4 inhibitors are conceptually attractive but will require especially rigorous pharmacological characterization.

### 5.5 Emerging inhibitors and next-generation modalities

PAD4 inhibitor development has expanded substantially in recent years. According to recent patent and translational reviews, the field now includes a wide range of scaffold classes beyond the early amidines and first-generation reversible probes, including benzimidazoles, azaindoles, imidazopyridines, thienopyrroles, fused bicyclic and tricyclic systems, selenium-containing heterocycles, and highly functionalized macrocycles (Aiken et al., 2025; Mansouri et al., 2024). Many of these newer compounds display nanomolar biochemical potency and improved absorption, distribution, metabolism, and excretion (ADME) properties relative to earlier inhibitors, indicating that PAD4 is no longer limited by target tractability alone.

Among the newer translational candidates, JBI-589 is one of the clearest examples of progress toward a clinically relevant PAD4 inhibitor. This orally available, non-covalent compound exhibits strong PAD4 selectivity, inhibits NET formation in mouse and human neutrophils, and has shown efficacy in mouse arthritis models (Gajendran et al., 2023). Such results are particularly significant because they connect improved chemical properties with *in vivo* disease modification in a therapeutically relevant context.

The same patent landscape suggests that JBI-1044 is among the most advanced current PAD4 programs, with activity reported in fibrosis, colitis, and cancer-related models and progress toward investigational new drug (IND)-enabling development (Aiken et al., 2025). Although public clinical data remain limited, the emergence of such candidates indicates that the PAD4 field has progressed beyond proof-of-concept pharmacology and into the realm of serious translational optimization. In this context, IND-enabling development refers to the package of preclinical studies - typically including pharmacokinetics, pharmacodynamics, toxicology, safety pharmacology, formulation, and manufacturing controls - required to support first-in-human clinical testing.

PAD4 targeting is also expanding beyond conventional small molecules. Functional anti-PAD4 antibodies have recently been identified that inhibit or modulate PAD4 activity through regulatory mechanisms distinct from classical active-site occupation (Zhou et al., 2024). These findings are important because they suggest that PAD4 function may be modulated allosterically or conformationally by larger biologics, potentially enabling new forms of selectivity or tissue targeting. Cyclic peptide modulators have also been developed and have provided mechanistic insight into PAD4 regulation and conformational control (Bertran et al., 2024). In parallel, a calcium-dependent allosteric binding pocket shared across PAD1-4 has recently been described, supporting the feasibility of non-covalent allosteric inhibition as a new design paradigm (Dakin et al., 2025). Together, these developments broaden the conceptual and technological space of PAD4 drug discovery.

### 5.6 Therapeutic applications across disease contexts

The therapeutic value of PAD4 inhibition depends on matching the inhibitor to the dominant pathogenic axis. In autoimmune disease, inhibition may reduce both citrullinated neoantigen generation and NET-mediated inflammatory amplification; in cardiovascular disease, it may limit NET-driven thrombosis and endothelial injury; in cancer, it may affect tumor plasticity, metastatic niche formation, macrophage immune restriction, and cancer-associated thrombosis (Lood et al., 2016; Knight et al., 2014; Curran et al., 2020; Chen et al., 2024; Döring et al., 2017; Zhu et al., 2022; Li J. et al., 2025; Seidlberger et al., 2026; Pitter et al., 2024; Causey et al., 2011; Gajendran et al., 2023). In infection and sepsis, however, PAD4 blockade is more complex because suppressing injurious NETosis may also compromise antimicrobial defense (Li et al., 2010; Liu et al., 2025; McDonald et al., 2017; Bonilha et al., 2025).

The optimal therapeutic application of PAD4 inhibitors depends on disease context—chronic autoimmune and cardiovascular diseases may benefit from sustained inhibition, whereas acute infection or sepsis demands careful timing to avoid compromising host defense.

## 6 Challenges and future directions

Despite substantial progress in PAD4 biology and inhibitor development, several major barriers continue to limit clinical translation. These challenges are scientific as well as pharmacological and must be addressed systematically if PAD4-targeted therapies are to move successfully into human medicine.

### 6.1 Isoform selectivity

One of the central challenges in PAD4 drug discovery is isoform selectivity. The PAD family shares a conserved catalytic architecture, making it difficult to inhibit PAD4 without affecting PAD1, PAD2, or PAD3 (Bicker and Thompson, 2013; Jones et al., 2009; Aiken et al., 2025; Knuckley et al., 2010). This matters because other PAD isoforms have distinct physiological roles, and off-target inhibition may produce unwanted biological effects. Early covalent inhibitors, although useful for proof-of-concept studies, were particularly limited in this regard (Causey et al., 2011; Luo et al., 2006b).

Recent advances in structure-guided medicinal chemistry have improved the ability to exploit subtle PAD4-specific features, including conformational preferences and residue-level differences in inhibitor binding (Aiken et al., 2025). The discovery of allosteric sites and conformationally sensitive regulatory surfaces may further expand opportunities for selectivity (Dakin et al., 2025; Zhou et al., 2024; Bertran et al., 2024). Future development should continue to prioritize isoform discrimination, because selectivity is likely to be essential for both safety and mechanistic precision.

### 6.2 Preserving host defense

PAD4 participates in antimicrobial immunity, particularly through NET formation (Li et al., 2010; Clark et al., 2018; Liu et al., 2025; McDonald et al., 2017). As a result, chronic or excessive inhibition could increase susceptibility to infection or impair pathogen control in selected settings. This issue is particularly important in diseases requiring long-term treatment, such as autoimmune disorders, chronic inflammatory states, or cancer-supportive therapy.

Importantly, the relationship between PAD4 inhibition and host defense is unlikely to be binary. Moderate or context-specific inhibition may suppress pathological NET overproduction without fully abolishing antimicrobial capacity. Moreover, the relevance of PAD4 may vary across pathogens, tissue sites, and inflammatory conditions. A major future priority will therefore be to define the therapeutic window of PAD4 modulation: how much inhibition is needed for benefit, in which diseases, and for how long, without producing clinically meaningful immune compromise.

### 6.3 Pharmacokinetics, tissue exposure, and route of administration

Many PAD4 inhibitors with strong biochemical activity have fallen short because of inadequate pharmacokinetic properties, poor oral bioavailability, short half-life, limited tissue penetration, or insufficient free drug exposure at sites of disease (Aiken et al., 2025; Gajendran et al., 2023). This is especially problematic in chronic diseases or in tissues where PAD4-driven pathology occurs in complex microenvironments, such as inflamed synovium, tumors, atherosclerotic plaques, or septic microvasculature.

Translational success will require not only potent compounds but also molecules with suitable absorption, distribution, metabolism, and elimination profiles. Oral bioavailability is particularly desirable for chronic inflammatory disease, whereas acute intravenous formulations may be more relevant in sepsis or myocardial infarction. Tissue-targeted delivery systems, nanoparticle formulations, or antibody-based approaches may help concentrate activity where PAD4 is most pathogenic and reduce systemic exposure. Such strategies may prove particularly valuable when the therapeutic index is narrow.

### 6.4 Biomarkers and patient stratification

A major translational bottleneck in the PAD4 field is the lack of validated biomarkers that reliably identify PAD4-driven disease biology and measure pharmacodynamic response. Several candidates have been proposed, including circulating citrullinated histones, NET-associated markers, anti-PAD4 antibodies, ACPAs, and broader citrullination signatures (Pollmann et al., 2012; Zhang G. et al., 2024; Döring et al., 2017; Mangold et al., 2015; Kijak-Boćkowska et al., 2025). However, none has yet been established as a fully validated biomarker platform for clinical development.

This gap matters because PAD4 is unlikely to be equally relevant in all patients with a given disease. In RA, PAD4-targeted therapy may be most effective in patients with strong ACPA responses, prominent NET signatures, or anti-PAD4 autoantibodies. In SLE, interferon-high, NET-rich phenotypes may be especially relevant. In cancer, PAD4 dependence may vary by tumor type, metastatic propensity, and thrombotic burden. Precision-medicine approaches that integrate PAD4 expression, citrullination patterns, immune profiling, and thromboinflammatory markers are therefore likely to be essential for patient selection and trial design.

### 6.5 Disease heterogeneity and context-specific biology

PAD4 biology is deeply context dependent. In some diseases, its dominant role lies in NET formation; in others, chromatin regulation, autoantigen generation, extracellular matrix remodeling, or immunothrombosis may be more important. Even within a single disease, the contribution of PAD4 may differ by disease stage, tissue compartment, inflammatory stimulus, and treatment background. This heterogeneity complicates both target validation and therapeutic strategy.

Future research should therefore move beyond viewing PAD4 as a uniform target and instead define disease-specific PAD4 dependence. This will require integrated approaches combining structural biology, single-cell analysis, spatial transcriptomics, proteomics of citrullinated substrates, and carefully designed pharmacological intervention studies. Such work will help determine not only whether PAD4 is involved in a disease, but how, when, and in which patient subsets it matters most.

### 6.6 Clinical trial design and combination therapy

The first successful PAD4-directed therapies are likely to emerge not simply from better inhibitors, but from better clinical strategy. Trial design should be grounded in disease biology, patient stratification, and the expected mechanism of benefit. In acute conditions such as thrombosis or sepsis, short-term PAD4 inhibition may be sufficient and may minimize safety risks. In chronic autoimmune disease, sustained treatment may require careful infection monitoring and pharmacodynamic surveillance. In oncology, PAD4 inhibitors may prove most effective in combination with anti-inflammatory, anticoagulant, cytotoxic, or immunomodulatory therapies.

Combination approaches may be most useful where PAD4 acts within broader inflammatory networks: with cytokine or B-cell-directed therapy in autoimmunity, antiplatelet or anticoagulant strategies in thromboinflammation, and immune checkpoint or anti-metastatic approaches in cancer. Such strategies could improve efficacy while reducing the dose required for PAD4 blockade.

### 6.7 Conclusions and perspectives

PAD4 has emerged as a central regulator at the interface of epigenetics, innate immunity, inflammation, thrombosis, and tissue remodeling. Through calcium-dependent citrullination of histone and non-histone substrates, PAD4 alters protein charge, chromatin structure, transcriptional output, extracellular matrix properties, and immunological antigenicity. These molecular activities enable PAD4 to influence diverse biological processes, but they also explain why dysregulated PAD4 activity can be pathogenic across a wide range of human diseases.

Across disease settings, PAD4 should be viewed as a mechanistic amplifier rather than as a disease-specific factor. Its recurring pathological roles are citrullinated neoantigen generation, NET-driven inflammatory and thrombotic injury, chromatin/transcriptional remodeling, extracellular matrix remodeling, and immune-context modulation. This integrated framework reduces repetition while preserving the major message: PAD4 becomes clinically important when citrullination is excessive, mislocalized, sustained, or coupled to unresolved inflammation.

These insights have established PAD4 as a compelling therapeutic target. The field has evolved from first-generation irreversible inhibitors such as Cl-amidine and F-amidine to selective reversible compounds such as GSK484 and GSK199, and further toward orally available translational candidates including JBI-589 and JBI-1044. In parallel, new strategies involving allosteric ligands, cyclic peptides, and functional antibodies are expanding the technological possibilities of PAD4 modulation. This rapid diversification of chemical and biological approaches underscores both the tractability of the target and the growing maturity of PAD4-directed drug discovery.

Nevertheless, enthusiasm must be matched by translational rigor. Several critical challenges remain unresolved, including isoform selectivity, pharmacokinetic optimization, biomarker validation, preservation of antimicrobial host defense, and identification of the patient populations most likely to benefit from treatment. The future of the field will depend on the integration of structural biology, medicinal chemistry, disease mechanism studies, and precision-medicine frameworks. It will also depend on a more refined understanding of when PAD4 is a disease driver, when it is merely a marker of inflammatory activation, and when its inhibition may carry unacceptable physiological cost.

In summary, PAD4 has emerged as a compelling target at the intersection of chromatin regulation, autoimmunity, thrombosis, and cancer biology. Its central position within these networks creates genuine therapeutic opportunity, but successful translation will depend on selective chemistry, careful biological positioning, and biomarker-guided clinical development. The next phase of the field should therefore focus less on proving that PAD4 matters and more on defining when, where, and in whom PAD4-directed intervention is most likely to deliver meaningful clinical benefit.
